# Subthreshold 577 nm micropulse laser treatment for central serous chorioretinopathy

**DOI:** 10.1371/journal.pone.0184112

**Published:** 2017-08-29

**Authors:** Ichiro Maruko, Hideki Koizumi, Taiji Hasegawa, Hisaya Arakawa, Tomohiro Iida

**Affiliations:** Department of Ophthalmology, Tokyo Women’s Medical University School of Medicine, Shinjuku, Tokyo, Japan; Massachusetts Eye & Ear Infirmary, Harvard Medical School, UNITED STATES

## Abstract

**Purpose:**

To compare the efficacy of the subthreshold micropulse laser (SML) to conventional laser (CL) in treating focal leakages of the retinal pigment epithelium (RPE) in the eyes with central serous chorioretinopathy (CSC).

**Methods:**

Twenty-nine eyes of 28 patients with CSC and typical focal leakage were treated with CL or SML. Both treatments were made with a 577 nm yellow laser (CL: NIDEK MC-500, SML: IRIDEX IQ577). The percentage of eyes with a complete resolution, the distance of the laser burns from the fovea, and injury of the RPE after treatment were studied.

**Results:**

A complete resolution was seen in 10 of 15 eyes (66.7%) after CL and 9 of 14 eyes (64.3%) after SML (*P* = 0.89). The average distance from the foveal center to the leakage point was 1282±596 μm for eyes treated with CL and 1271±993 μm for eyes treated with SML (*P* = 0.4). Only three eyes treated with SML had treatment sites within 500 μm of the fovea. RPE damage determined by fundus autofluorescence was found in all eyes treated with CL and only one eye treated with SML (*P*<.01).

**Conclusions:**

SML achieved equivalent therapeutic effects as CL but without RPE damage in eyes with CSC.

## Introduction

Central serous chorioretinopathy (CSC) is characterized by an idiopathic serous retinal detachment (SRD) in the posterior pole associated with focal or diffuse leakage from the retinal pigment epithelium (RPE).[1] Some of the SRDs resolve spontaneously, and the vision is generally good. However, recurrent or persistent cases of CSC can cause visual reductions.[2–4] Although laser treatment to the focal leakage sites is still a standard therapy for such cases,[5–7] this method is difficult to implement in eyes with leakage sites within or very close to the foveal avascular zone (FAZ). The difficulty is because conventional laser (CL) treatments usually leave atrophic scars of varying sizes on the RPE.

Recently, subthreshold laser treatments were reported to be effective in treating eyes with CSCs.[8–12] Several reports have compared the efficacies of the different treatments for CSC including subthreshold laser treatments, CL, photodynamic therapy, and intravitreal injections of anti-vascular endothelial growth factor.[13,14] However, there is no study comparing CL treatments to subthreshold micropulse laser (SML) treatments on the complete resolution of the CSC and RPE damages.

Thus, the purpose of this study was to compare the efficacy of SML to CL for the treatment of focal leakages of the RPE in eyes with CSC.

## Materials and methods

This was a retrospective study conducted according to the tenets of the Declaration of Helsinki. The Institutional Review Board of the Tokyo Women’s Medical University School of Medicine approved the study which included the use of optical coherence tomography (OCT) and fundus autofluorescence (FAF) on eyes with macular and retinal disorders, observational study of age-related macular degeneration, and similar disorders including CSC. In our institution, a consent form is not required for a retrospective study.

Twenty-nine eyes of twenty-eight patients (20 men, 8 women; average age, 48.4 years) with CSC and typical focal leakage of more than 3-months duration were studied. The eyes were treated with CL or SML from November 2015 to October 2016. Because SML therapy was introduced in our hospital in May 2016, CL was performed until April 2016, and SML was conducted from May 2016.

The clinical examinations used to diagnose CSC included measurements of the best-corrected visual acuity (BCVA), slit-lamp biomicroscopy with and without a contact lens, indirect ophthalmoscopy, and digital fluorescein and indocyanine green angiography (HRA2, Heidelberg Engineering, Heidelberg, Germany). The BCVA was measured with a Japanese standard visual acuity chart, and the decimal BCVA was converted to the logarithm of the minimum angle of resolution (logMAR) units for the statistical analyses.

All eyes were examined by swept source OCT (DRI-OCT Atlantis, Topcon, Tokyo, Japan) to evaluate the SRD. The central retinal thickness and subfoveal choroidal thickness were measured using the caliper tool in the OCT software before and after the treatment. The thicknesses of the retina was defined as the distance from the inner limiting membrane to the inner RPE surface including SRD, and the choroidal thickness as the distance between the outer RPE surface and the inner scleral surface.

CSC was diagnosed to be present if the eye had subretinal fluid involving the macula that was associated with idiopathic leaks from the RPE detected during fluorescein angiography. Only eyes with CSC and typical focal leakage of more than 3-months duration were included in this study. Thus, eyes with the chronic type of CSC with diffuse leakage were excluded because of difficulty in identifying the treatment sites. The distance between the leakage point and the foveal center was measured in the early phase of fluorescein angiography. Indocyanine green angiography was used to confirm the presence of choroidal vascular hyperpermeability, and to rule out the presence of choroidal neovascularization including polypoidal choroidal vasculopathy.

All patients had a reduction of the visual acuity, and they agreed to the laser treatment after being informed of the risks and benefits. The duration of the SRD was estimated from the patient's recall of the onset of the visual symptoms.

CL was performed with a 577 nm yellow laser (NIDEK MC-500, Nidek, Gamagori, Japan) with a 200 μm spot diameter, a 0.20 sec duration, and 60–80 mW power. The endpoint of CL photocoagulation was the production of a slight graying of the RPE. SML photocoagulation was performed with a 577 nm micropulse yellow laser (IRIDEX IQ577) with a 200 μm spot diameter, a 0.20 sec duration with 15% duty cycle (D/C), and 140–200 mW power. The actual laser power for the SML treatment was determined by the visible laser scar of a trial photocoagulation created with continuous wave laser energy for 0.1 s with a diameter of 200 μm outside the vascular arcade without a SRD. About 70–100 mW power with a continuous wave laser was enough to develop a graying of the RPE. Thereafter, laser spots were applied using a 15% duty cycle micropulse mode at 200% of the threshold energy for 0.20 sec which delivered 60% of the threshold energy.[15] The number of SML applications in one session was limited to 10 shots because the laser scars were not visible. The RPE changes after the treatment with CL or SML were also analyzed by FAF (CX-1 MYD/NM, Canon, Japan).

The percentage of eyes with a complete resolution, the distance between the leakage points and the foveal center, the frequency of the treatments, the changes in the BCVA, the subfoveal choroidal thickness, and the RPE damage after treatment were studied.

All *P*-values were two-sided and a *P* <0.05 was considered statically significant in Mann-Whitney *U* test and the Wilcoxon signed-rank test. All statistical analyses were performed with EZR (Saitama Medical Center, Jichi Medical University, Saitama, Japan), which is a graphical user interface for R (The R Foundation for Statistical Computing, Vienna, Austria).[16] More precisely, it is a modified version of R commander designed to add statistical functions frequently used in biostatistics.

## Results

CL treatment was performed on 15 eyes of 14 patients (9 men, 5 women; average age, 49.9 years) from November 2015 to April 2016, and SML treatment was performed on 14 eyes of 14 patients (11 men, 3 women; average age, 46.9 years) from May 2016 to October 2016. There were no significant differences in the duration of the symptoms between the two groups. The baseline characteristics, the resolution of the SRD, the number of treatments, the BCVA before and after treatment, the central retinal thickness before and after treatment, the subfoveal choroidal thickness before and after treatment are summarized in Table 1.

**Table 1:** Baseline background and post treatment characteristics in the eyes with conventional laser and subthreshold micropulse laser treatments.

Treatment	n	Symptom (M)	Complete Resolution	F/U (M)	Treatment Frequency	BCVA (snellen)		CRT ± SD (μm)		SCT ± SD (μm)	
						Before	After	Before	After	Before	After
CL	15	3.1	10 (66.7%)	3.4	1.13	0.92	0.94	338±135	173±37	433±89	423±91
SML	14	2.4	9 (64.3%)	2.2	1.36	0.96	0.94	328±129	192±64	411±106	420±106
Diff (P-value)		no (0.28)	no (0.89)	no (0.74)	no (0.31)	no(0.45)	no(0.45)	no (0.83)	no (0.78)	no (0.56)	no (0.98)

BCVA = best-corrected visual acuity

SD = standard deviation

CL = conventional laser

SML = subthreshold micropulse laser

Symptom (M) = subjective symptom duration (months)

F/U (M) = follow-up periods or time to resolution (months)

CRT = central retinal thickness

SCT = subfoveal choroidal thickness

Diff = significant differences between CL and SML

The SRD was resolved in 10 of 15 eyes (66.7%) after CL and 9 of 14 eyes (64.3%) after SML (*P* = 0.89). The time to resolution of the SRD was 3.3 months after CL and 1.9 months after SML (*P* = 0.16, Mann Whitney U-test). The average follow-up period was 3.4 months after CL and 2.2 months after SML (*P* = 0.74, Mann Whitney U-test). The average number of treatments was 1.13 for CL and 1.34 for SML (*P* = 0.31, Mann Whitney U-test).

The mean decimal BCVA was 0.92 at the baseline and 0.95 after CL. For the SML group, the mean decimal BCVA was 0.96 at the baseline and 0.94 after the treatment. None of these differences in the BCVA was significant (both CL and SML, *P* >0.1, Wilcoxon signed-rank test). The mean central retinal thickness before treatment was 338 ± 135 μm in the CL group and 328 ± 129 μm in the SML group (*P* = 0.83, Mann Whitney U-test). The mean central retinal thickness after treatment was 173 ± 37 μm in the CL group and 192 ± 64 μm in the SML group (*P* = 0.78, Mann Whitney U-test). There was a significant difference between the central retinal thickness before and after treatment in both the CL and SML groups (both *P* <0.01, Wilcoxon signed-rank test). The mean subfoveal choroidal thickness before treatment was 433 ± 89 μm in the CL group and 411 ± 106 μm in the SML group (*P* = 0.56, Mann Whitney U-test). The mean subfoveal choroidal thickness after treatment was 423 ± 91 μm in the CL group and 420 ± 106 μm in the SML group (*P* = 0.98, Mann Whitney U-test). There was no significant difference between the choroidal thicknesses before and after treatment in both CL (*P* = 0.13, Wilcoxon signed-rank test) and SML (*P* = 0.45, Wilcoxon signed-rank test) groups.

The mean distance between the leakage site and the foveal center was 1282 ± 596 μm (range, 539–2498 μm) for the CL treatment group and 1271 ± 993 μm (range, 310–3498 μm) for the SML treatment group (*P* = 0.4, Mann Whitney U-test). There were 4 eyes (26.7%) treated with CL and 7 eyes (50.0%) treated with SML where the leakage sites were within 1000 μm of the foveal center. Three of 7 eyes in the SML group had treatment sites within 500 μm of the fovea. (Table 2)

**Table 2:** Distance between leak point and foveal center in the eyes with conventional laser and subthreshold micropulse laser treatments.

Treatment	Distance ± SD	500–1000 μm	≤500 μm
CL	1282±596	4	0
SML	1271±993	4	3

Distance = mean distance between leak point and foveal center

SD = standard deviation

CL = conventional laser

SML = subthreshold micropulse laser

**Fig 1:**
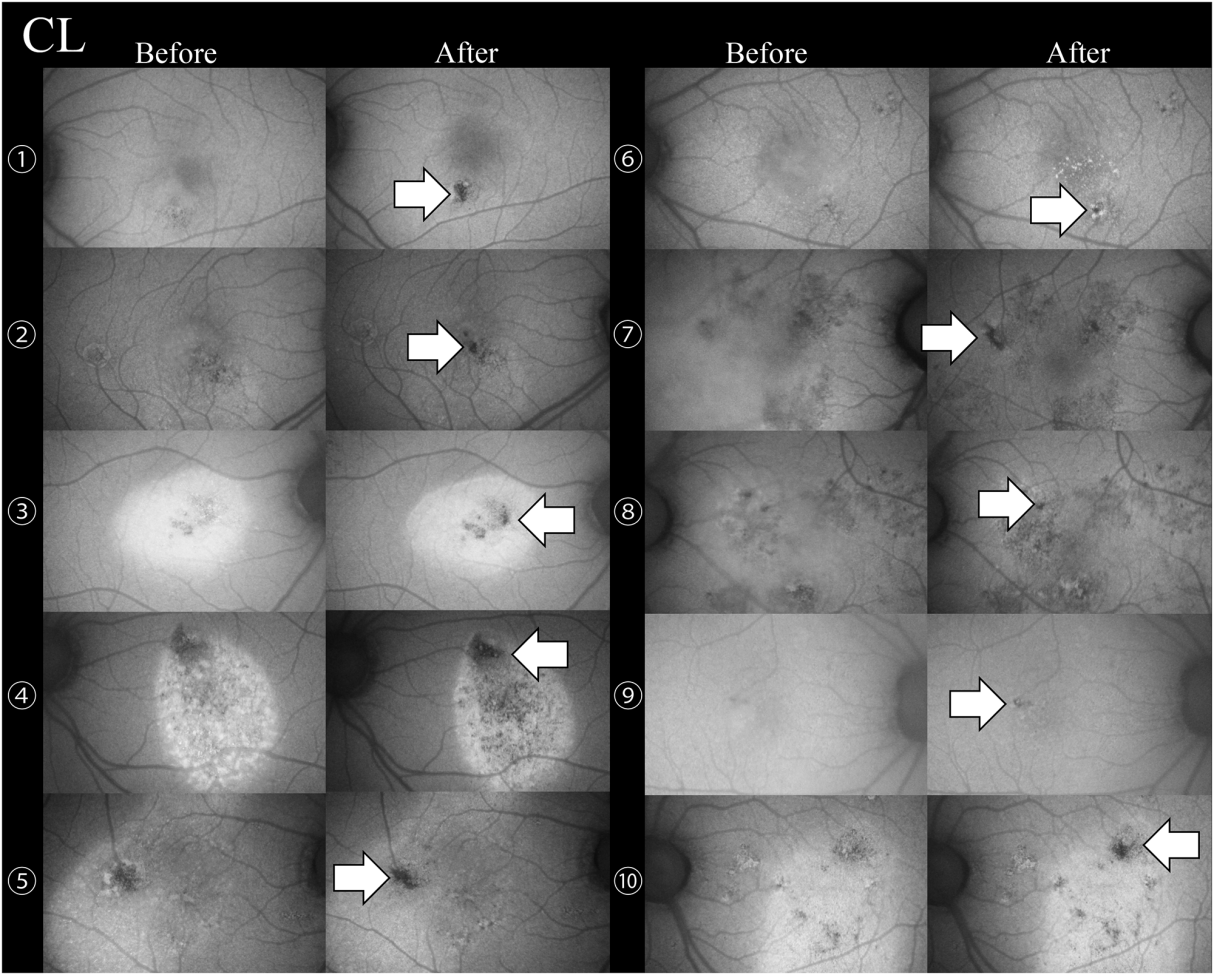
Changes in the fundus autofluorescence before and after conventional laser (CL) treatment in 10 eyes with central serous chorioretinopathy. Retinal pigment epithelial damage was observed in 10 of the 10 eyes (white arrows) after the CL treatment.

**Fig 2:**
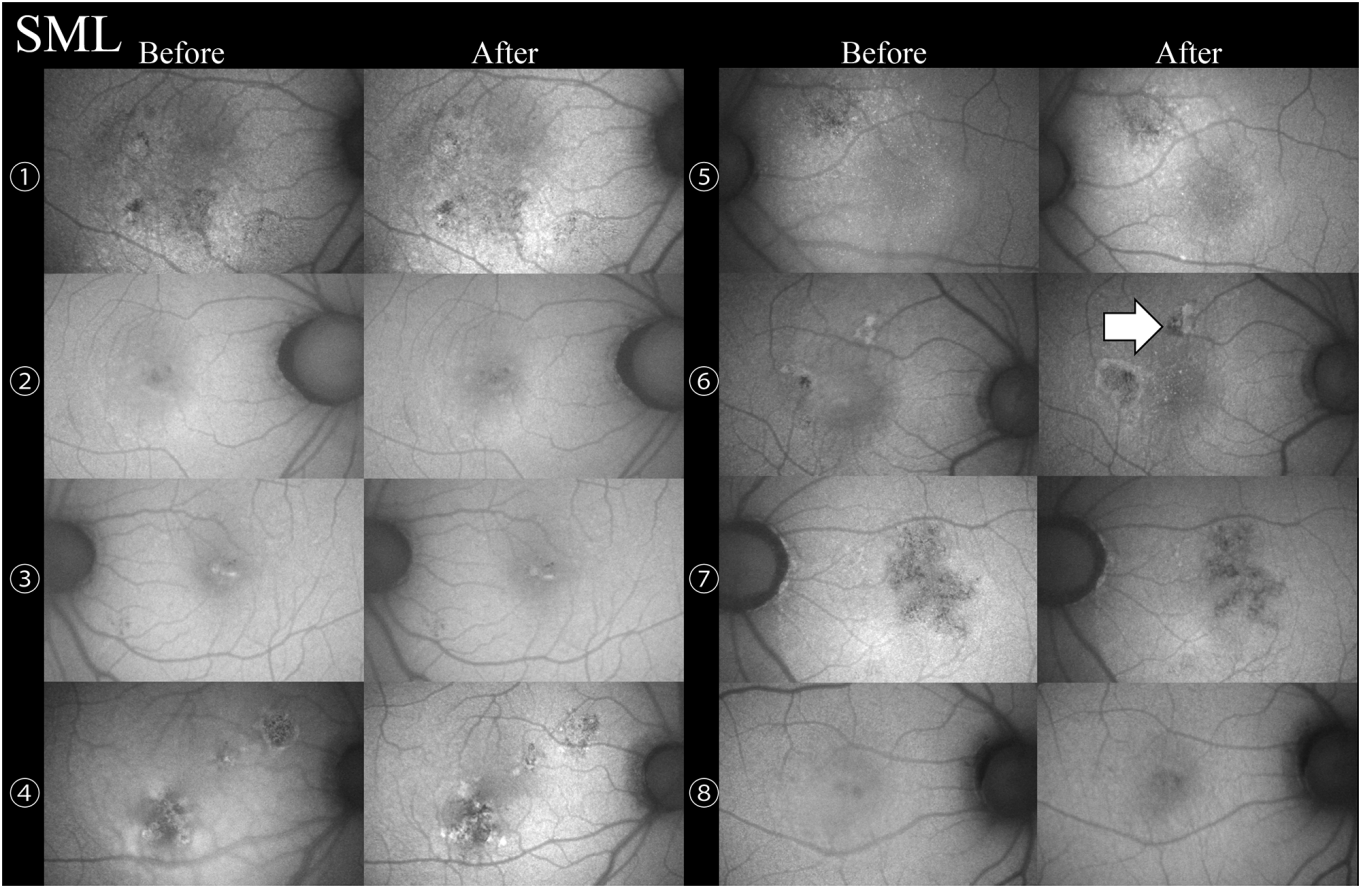
Changes on fundus autofluorescence before and after subthreshold micropulse laser (SML) treatment in 8 eyes with central serous chorioretinopathy. Retinal pigment epithelial damage was present in only 1 of 8 eyes (white arrow) after the SML treatment.

The changes in the RPE were evaluated by FAF before and after treatment in the eyes with complete resolution excluding one eye of a SML cases without FAF images before treatment. RPE damage was observed in 10 of 10 eyes (Fig 1) in the CL treated group and 1 of 9 eyes (12.5%, Fig 2) in the SML treated group (P <0.01). A 32-year-old man with SRD due to CSC was treated twice with SML, and the second session was performed with the maximum number of 200 mW pulses (D/C 15%) with the same SML parameters except for the laser power. In only one case, FAF showed that the RPE was damaged at the sites of the second SML session (Fig 2, Case 6).

## Discussion

Our findings showed that SML treatment was almost as effective as CL treatment for the eyes with CSC. Although CL treatment left permanents scar in all eyes, SML caused almost no RPE damage as determined by FAF in the eyes with a complete resolution.

CL photocoagulation is still one of the standard therapies for CSC, however it is not possible to treat leakages within or close to the FAZ because an RPE scar remains after the treatment. Photodynamic therapy (PDT) is an option not only for chronic CSC with diffuse leakage but also for acute CSC with leakage within or close to the FAZ.[17–20] PDT has not been approved for CSC, and it is not a standard therapy. In contrast, SML is expected to be accepted for CSC with leakage within the FAZ because SML does not cause RPE scars. Elhamid et al.[8] reported on the efficacy of SML in 15 eyes with CSC including 9 eyes with leakage within the FAZ. They had a complete resolution in 73% of the eyes. In our study, we successfully treated 3 eyes with leakage within 500 μm of the fovea although we did not treat eyes with leakages within the FAZ. These results suggest that SML can be safely used for CSC even on leakages close to the foveal center. However, further studies are needed to determine whether SML treatment within the FAZ should be considered.

CL usually uses continuous-wave lasers for the photocoagulation, and the heat it generates affects not only the targeted RPE cells but also the surrounding areas. This then causes RPE scars larger than expected, and the expansion of laser scars is called “atrophic creep” after laser photocoagulation. On the other hand, SML generates less heat. This then leads to less damage of the targeted RPE and the surrounding areas than CL.

RPE damage was not observed in the cases with SML treatment except for one case. We treated a recurrent case with a second session of SML with 200 mW (D/C 15%), and RPE scars developed at the sites of the applications. Thus, there is a limit in the use of SML. It is necessary to be careful even for SML treatments especially the laser power.

Lanzetta et al.[9] and Chen et al.[10] also used SML with an 810-nm laser, and they reported a complete resolution in 71% and 55% respectively. Yadav et al.[11] treated 15 eyes with chronic CSC with a 577-nm laser, but they reported only 40% of the eyes had a complete resolution. Sholz et al.[12] in their large comparison study between SML and half-dose PDT for chronic CSC reported a complete resolution in 36% of the SML group with a 577-nm laser during 6 weeks follow-up period. Our results were better with a complete resolution in about 65%, which may because our cases include the eyes without the diffuse leakage but only with the typical focal leakage.

There have been reports on the benefits of SML treatment compared with alternative treatments.[13, 14] The effects of SML on chronic CSC should be compared with that of PDT. In fact, Sholz et al. described the superiority of SML to half-dose PDT for chronic CSC. In another report, Özmert et al.[21] reported that the results of SML was as good as that of PDT for chronic CSC with durations of more than 6 months. Koss et al.[22] reported that SML reduced the leakage more than intravitreal bevacizumab injections. SML might be the new standard treatment for CSC.

This retrospective study had several weaknesses including the small sample size and short follow-up periods. In addition, it was difficult to completely determine whether the results were due to the treatment or spontaneous resolution although it was unlikely that cases that did not recover spontaneously for more than 3 months might recover at this high rate in both groups. In addition, this was not a truly comparative study because the study time periods were different. There might also have been a selection bias for the eyes with leakages closer to fovea because no RPE scar remained in most of the SML cases.

In conclusion, the results showed that SML was as effective as CL in eyes with typical CSC. In addition, RPE damage was found significantly more frequently after CL than after SML. SML treatment within FAZ may also be considered as the results. However, it will be necessary to obtain data from a larger number of patients including those with chronic CSC to demonstrate the complete safety of SML especially for eyes with leakages close to the FAZ.

## Supporting information

S1 Table Baseline characteristics and changes before and after treatment in all data. (DOCX)
