# Supplementary material for: Subthreshold 577 nm micropulse laser treatment for central serous chorioretinopathy
Source: PLoS One. 2017 Aug 29;12(8):e0184112. doi: 10.1371/journal.pone.0184112 (PMC5574536; doi:10.1371/journal.pone.0184112)
Supplement: S1 Table — (DOCX) [file pone.0184112.s001.docx]

| S1 Table. Baseline Characteristics and Changes before and after treatment | | | | | | | | | | | | | |
| --- | --- | --- | --- | --- | --- | --- | --- | --- | --- | --- | --- | --- | --- |
| patient # | age | gender | Eye | Symptom (M) | F/U (D) | Treatment Frequency | BCVA (snellen) | | CRT (µm) | | SCT (µm) | | Distance (µm) |
| CL |  |  |  |  |  |  | before | after | bofore | after | bofore | after |  |
| 1 | 52 | M | OD | 3 | 12.2 | 2 | 1.2 | 1.2 | 183 | 193 | 295 | 304 | 1690 |
| 2 | 46 | M | OD | 2 | 7.9 | 1 | 1.2 | 1.2 | 337 | 155 | 435 | 389 | 2369 |
| 3 | 67 | F | OD | 6 | 2.7 | 1 | 0.5 | 0.4 | 162 | 172 | 428 | 431 | 1574 |
| 4 | 45 | M | OD | 3 | 0.9 | 1 | 1.2 | 1.2 | 622 | 141 | 400 | 403 | 1351 |
|  |  |  | OS | 3 | 2.8 | 1 | 1.2 | 1.2 | 552 | 179 | 302 | 283 | 1098 |
| 5 | 75 | F | OD | 3 | 1.0 | 1 | 0.9 | 1.2 | 337 | 183 | 499 | 516 | 624 |
| 6 | 47 | M | OS | 1 | 0.9 | 1 | 0.9 | 0.9 | 151 | 112 | 611 | 587 | 539 |
| 7 | 29 | M | OS | 4 | 6.1 | 1 | 0.5 | 1.2 | 397 | 197 | 506 | 509 | 2498 |
| 8 | 52 | M | OS | 1 | 1.4 | 1 | 1.2 | 1.2 | 443 | 225 | 513 | 481 | 710 |
| 9 | 38 | M | OS | 6 | 0.7 | 1 | 0.7 | 0.7 | 257 | 116 | 386 | 367 | 1502 |
| 10 | 50 | F | OS | 1 | 0.7 | 1 | 1.2 | 1.2 | 376 | 179 | 527 | 544 | 1299 |
| 11 | 44 | M | OD | 6 | 8.9 | 2 | 1.2 | 1.2 | 309 | 232 | 456 | 459 | 546 |
| 12 | 53 | F | OD | 2 | 1.2 | 1 | 0.6 | 0.4 | 228 | 134 | 351 | 339 | 1354 |
| 13 | 40 | M | OS | 5 | 0.9 | 1 | 0.8 | 0.8 | 334 | 155 | 449 | 389 | 1000 |
| 14 | 61 | F | OD | 1 | 11.2 | 1 | 1.2 | 1.2 | 380 | 218 | 344 | 339 | 1075 |
|  |  |  |  |  |  |  |  |  |  |  |  |  |  |
| SML |  |  |  |  |  |  |  |  |  |  |  |  |  |
| 1 | 57 | M | OS | 3 | 102 | 2 | 1.2 | 1.2 | 299 | 285 | 344 | 339 | 636 |
| 2 | 36 | M | OD | 2 | 33 | 1 | 0.2 | 0.2 | 158 | 134 | 632 | 622 | 1948 |
| 3 | 36 | M | OS | 4 | 32 | 1 | 1.2 | 1.2 | 239 | 183 | 506 | 509 | 310 |
| 4 | 41 | M | OS | 1 | 42 | 1 | 1.2 | 1.2 | 404 | 127 | 400 | 382 | 3498 |
| 5 | 57 | M | OS | 3 | 45 | 1 | 1.2 | 1.2 | 183 | 155 | 372 | 375 | 1169 |
| 6 | 64 | M | OD | 3 | 112 | 1 | 1.2 | 1.2 | 369 | 162 | 372 | 396 | 646 |
| 7 | 49 | F | OD | 6 | 35 | 1 | 0.9 | 0.8 | 351 | 246 | 344 | 346 | 2228 |
| 8 | 32 | M | OD | 1 | 70 | 2 | 1.2 | 0.7 | 320 | 134 | 464 | 481 | 1033 |
| 9 | 37 | M | OD | 1 | 42 | 1 | 1.2 | 1.2 | 520 | 151 | 407 | 481 | 431 |
| 10 | 51 | M | OD | 1 | 91 | 2 | 1 | 1 | 235 | 320 | 435 | 431 | 1158 |
| 11 | 31 | M | OD | 6 | 84 | 1 | 0.8 | 1.2 | 218 | 172 | 541 | 544 | 488 |
| 12 | 75 | F | OD | 1 | 34 | 1 | 0.8 | 0.7 | 253 | 179 | 176 | 184 | 867 |
| 13 | 47 | F | OD | 1 | 140 | 3 | 1.2 | 1.2 | 450 | 281 | 379 | 424 | 493 |
| 14 | 44 | M | OD | 1 | 49 | 1 | 1.2 | 1.2 | 597 | 158 | 386 | 367 | 2885 |
|  |  |  |  |  |  |  |  |  |  |  |  |  |  |
| BCVA = best-corrected visual acuity | | | | |  |  |  |  |  |  |  |  |  |
| SD = standard deviation | | |  |  |  |  |  |  |  |  |  |  |  |
| CL = conventional laser | | | |  |  |  |  |  |  |  |  |  |  |
| SML = subthreshold micropulse laser | | | |  |  |  |  |  |  |  |  |  |  |
| Symptom (M) = subjective symptom duration (days) | | | | | |  |  |  |  |  |  |  |  |
| F/U (D) = follow-up periods or time to resolution (months) | | | | | |  |  |  |  |  |  |  |  |
| CRT = central retinal thickness | | | |  |  |  |  |  |  |  |  |  |  |
| SCT = subfoveal choroidal thickness | | | |  |  |  |  |  |  |  |  |  |  |
| Distance = mean distance between leak point and foveal center | | | | | |  |  |  |  |  |  |  |  |
